# Urbanisation at Multiple Scales Is Associated with Larger Size and Higher Fecundity of an Orb-Weaving Spider

**DOI:** 10.1371/journal.pone.0105480

**Published:** 2014-08-20

**Authors:** Elizabeth C. Lowe, Shawn M. Wilder, Dieter F. Hochuli

**Affiliations:** School of Biological Sciences, The University of Sydney, Sydney, New South Wales, Australia; University of Sydney, Australia

## Abstract

Urbanisation modifies landscapes at multiple scales, impacting the local climate and changing the extent and quality of natural habitats. These habitat modifications significantly alter species distributions and can result in increased abundance of select species which are able to exploit novel ecosystems. We examined the effect of urbanisation at local and landscape scales on the body size, lipid reserves and ovary weight of *Nephila plumipes*, an orb weaving spider commonly found in both urban and natural landscapes. Habitat variables at landscape, local and microhabitat scales were integrated to create a series of indexes that quantified the degree of urbanisation at each site. Spider size was negatively associated with vegetation cover at a landscape scale, and positively associated with hard surfaces and anthropogenic disturbance on a local and microhabitat scale. Ovary weight increased in higher socioeconomic areas and was positively associated with hard surfaces and leaf litter at a local scale. The larger size and increased reproductive capacity of *N.plumipes* in urban areas show that some species benefit from the habitat changes associated with urbanisation. Our results also highlight the importance of incorporating environmental variables from multiple scales when quantifying species responses to landscape modification.

## Introduction

Urbanisation modifies landscapes and degrades habitats in order to support increasing human populations in cities around the world [1]. This alteration of natural areas tends to decrease diversity while increasing densities of select species [2], resulting in biological communities with novel species interactions [3] and altered ecosystem function [4]. Many species are excluded from these novel environments but some species can live and even thrive in cities, becoming urban exploiters [5]. The probability of a species becoming an urban exploiter often depends on plasticity or pre-adaptation [6], but there are also examples of changes in behaviour [7], morphology [8] and life history [9] as a result of adaptation to novel environments. While the effects of urbanisation on wildlife have most often been studied in birds [10], invertebrates provide an ideal study system in which to examine the fitness consequences of urbanisation at both small scales and across landscapes [11].

Although biodiversity responds to urbanisation at multiple spatial scales, many studies investigating its effect on wildlife draw comparisons between urban and rural areas but fail to quantify the level of urbanisation or incorporate broad scale environmental variables [10]. A gradient from the centre of the city to outlying natural areas must incorporate multiple sites from each landscape type in order to account for the high levels of heterogeneity in the urban matrix [12]. For the purpose of this study, landscape scale refers to environmental and socioeconomic factors in the Sydney region. In the context of urbanisation, this relates to fragmentation of habitats, increases in housing and population densities and changes to the surrounding land cover from predominantly vegetation to a matrix of hard surfaces, housing, industry and parklands. These broad scale increases in impermeable surfaces and decrease in vegetation can lead to changes in climate [13] which, similarly to climate change, have been shown to alter species distributions [14]. Local scale refers to the environment in the surrounding area, for example within the boundaries of a suburb or national park. At a microhabitat scale, species in urban areas are directly influenced by decreases in habitat complexity [15] and changes in species interactions [16]. Identifying the scale at which anthropogenic changes affect animals can aid in managing the impact of urbanisation on biodiversity and ecosystem health.

While large native predators are often lost from urban environments as a result of habitat loss and conflict with humans [17], some generalist predators benefit from urbanisation [18], [19]. Changes in predator abundance can lead to the uncoupling of trophic interactions in urban areas [3], [20] leading to further decreases in biodiversity and ecosystem health. Spiders are a useful taxa in which to study the effect of urbanisation on predators as they can have large effects on food webs and ecosystem traits including herbivore abundance, plant community composition and nutrient cycling [21]. By studying the responses of spiders to anthropogenic change we gain a better understanding of how to maintain trophic interactions and biodiversity in urban systems.

Previous studies concerning the species richness and abundance of spiders in response to anthropogenic disturbance show varied responses. Some studies found no response to urbanisation [22] while others found an increase in species diversity [23] or an increase in total abundance as result of select species becoming urban exploiters [24], [25]. This variation is likely a result of different sampling methods [26], environmental variation between cities [27] and differing classifications of urban landscape traits [10].

We tested how urbanisation affects the growth and reproduction of the golden orb weaving spider, *Nephila plumipes. Nephila* are common spiders in the Asia Pacific region [28] and *N.plumipes* is abundant along the east coast of Australia [29]. They build semi-permanent webs which, once mature, they remain in for the duration of their life span. This sedentary lifestyle, along with the fact that spider size and condition can be strongly influenced by their environment [30], [31], makes *N.plumipes* an ideal species in which to study the effects of microhabitat on condition. We examined associations between measures of urbanisation and the size, lipid reserves and egg production of spiders at multiple scales along a gradient from city to continuous bush land in Sydney, Australia. The urban heat island effect and habitat fragmentation have both been shown to increase abundance of some herbivorous insects in urban areas [11], [32]. Spider development and fecundity can be strongly influenced by temperature (see [30]) and diet [31], therefore we predicted that *N.plumipes* size and condition would increase at higher levels of urbanisation. We also predicted that urbanisation would have the largest effect on *N.plumipes* morphology at a microhabitat scale, as spiders often respond to fine scale environmental modification [33], [34].

## Methods

### a) Study sites

Sydney is the largest city in Australia, supporting 4.39 million people over an area of 12,368 Km^2^ [35]. Study sites from within Sydney’s urban matrix were classified into three coarse landscape types; recreational parks and gardens (n = 7), remnant patches (n = 10) and continuous bushland (n = 3) (Table S1). Urban parks are characterised as small to medium sized fragments (ranging from 0.04 km^2^ to 0.7 km^2^) surrounded by housing with manicured garden beds and little to no native vegetation. The predominant land cover of these fragments is grass, non-native vegetation, buildings and hard surfaces. The urban remnant sites are fragmented patches of native vegetation ranging from 0.08 km^2^ to 1.6 km^2^ which are isolated from other patches of native vegetation and are now predominantly surrounded by housing. The continuous bushland sites consist mostly of native vegetation and are connected to large natural areas of bushland.

### b) Spider collection

The collection of spiders occurred between April and June 2012 for all sites, at which time a majority of the female spiders were mature. For each female spider, the size of the web’s capture area and height of the web above ground were recorded. A total of 222 spiders were collected (excluding males and immature females) with the number of spiders collected from each site ranging from 2–25, and an average of 11 spiders per site (Table S1). Spiders were collected as they were encountered, in rare cases this was all of the spiders in a site (for the small parks).

Spiders were collected with permission from the New South Wales National Parks and Wildlife Service (Scientific license #SL100915), the Royal Botanic Gardens and the following local councils; Manly, North Sydney, Ku-Ring-Gai, Willoughby, Warringah, Wolli Creek, Kogarah, Sydney City. *N. plumipes* is abundant throughout the region and a maximum of 25 individuals were removed from each site. Ethics permission is not required for invertebrates under the New South Wales Animal Research Act 1985.

### c) Habitat assessments

We measured habitat characteristics at three spatial scales: landscape, local and microhabitat. At a landscape scale, we measured the percentage land cover (vegetation, grass, hard surfaces and water) over a 1 km radius (centred in the area of the site where the majority of the spiders were found). The distance of each site to the following prominent landscape features; the central business district (CBD), permanent water, the coast, parkland and bushland, were calculated using Google Earth and Image J. The suburb demographics used for each site were: population density, housing density and average weekly income [35].

At a local scale, the site refers to the area within the boundary of each park, remnant patch or area of national park. For each site, the percentage land cover (vegetation, grass, water, hard surfaces and buildings), perimeter, area and shape were calculated using Image J and Google Earth.

At a microhabitat scale, six habitat characteristics (ground vegetation cover, shrub canopy cover, tree canopy cover, tree density, leaf litter and anthropogenic structures) were scored from 0–3 to indicate the habitat complexity for each spider (adapted from [36]). A high score for the summed habitat characteristics of each individual indicates a microhabitat with a high level of habitat complexity and a low level of disturbance. The percentage land cover (vegetation, grass, water, hard surfaces) over a 10 m radius was also calculated using Image J and Google Earth. The proximity of individual spiders to landscape features (open space, hard surfaces, buildings, site edge and water) was determined using the GPS locations of each spider in Google Earth. For each individual we also quantified the following; number of kleptoparasites in the web, number of conspecifics within a 5 m radius (as a measure of aggregation sizes) and number of prey items stored in the web.

### d) Morphological measurements

A range of morphological traits were measured for each spider to determine size and give multiple measures of condition. Size was quantified through the measurement of both hard (leg length, tibia length and carapace length/width) and soft (abdomen length, width and height) structures. Tibia length of the first leg was used as a measure of body size [37]. The ratio of weight to tibia length was used as a measure of body condition as the size of hard structures is constant in mature individuals while weight shows substantial variation depending on prey capture rates and reproductive state [37]. Other measures of body condition (e.g. the residuals of the weight/tibia ratio) yielded qualitatively similar patterns.

Lipid weight and ovary weight were determined for a subset of spiders as a more direct measure of nutrient reserves and egg production. A chloroform extraction protocol (adapted from Wilder and Rypstra [38]) was used to determine lipid weight of spiders. However, as the bodies of some spiders ruptured during drying, lipid weight estimates for the remaining spiders were calculated using a regression of abdomen volume and extracted lipid weight (n = 106, r^2^ = 0.20, lipid = 3E−05*volume+0.02). Ovary weight was measured by dissecting 29 individuals and was predicted for the remaining individuals using a regression of ovary weight against abdomen volume (n = 29, r^2^ = 0.62, ovary weight = 2E−4*volume-0.03).

### e) Statistics

Principal components analyses (PCA) were conducted using SPSS to combine environmental variables from a landscape (12 variables), local (8 variables) and microhabitat scale (15 variables). The resulting components from the three PCAs were used to represent different elements of urbanisation (Table S2). The first through fourth axes of these PCAs were compared to morphological measures (site average at local and landscape scales and individual measures at the microhabitat scale) using Pearson’s correlations. Differences between the morphology of spiders in different landscape types (bush, remnant or parks) were calculated using ANOVA’s (data available in Table S3).

## Results

### a) PCAs integrating habitat variables at landscape, local and microhabitat scales

The PCA combining 12 landscape scale variables resulted in four components (for the percentage variance explained by each component see Table S2). The first component was negatively associated with vegetation cover and distance to CBD, and positively associated with hard surface cover, housing density, population density and distance to bushland. As all of these landscape variable associations reflect urban characteristics, this first component was used as an index reflecting the degree of urbanisation at each site. The second component, which was associated with suburb house hold income and proximity to parklands, was used as an indication of the socioeconomics of each site. The third component did not combine any relevant variables but the fourth (combining distance to coast and percentage water over 1 km radius) was used to represent an association with the coast.

We derived two components from the PCA for the local scale which combined eight site variables (Table S2). The first component combined all land cover variables excluding water and was used to reflect urbanisation. The second component concerned habitat fragmentation and was associated with larger and less convoluted sites.

The PCA for the microhabitat scale combined 15 variables and resulted in 5 components (Table S2). The first component reflected more natural habitats with a positive association with increased distance from anthropogenic features (e.g. hard surface or site edge) and the percentage land cover of water. A negative association with hard surfaces and man-made structures and a positive association with percentage vegetation, indicating habitats with less disturbance, were represented in component two. The third component reflected increased ground cover complexity (less grass and more leaf litter) and the forth was associated with increased habitat complexity.

### b) Variation in size and condition

Tibia length ranged from 6.91 mm to 14.03 mm, with an average of 10.74±1.37 mm and was significantly different between sites (F_19,202_ = 2.59, *p*<0.01). Weight was also significantly different between sites (F_19,202_ = 2.39, *p*<0.01) and ranged from 0.13 g to 2.63 g with an average of 0.97±0.45 g. The predicted lipid weights calculated for spiders ranged from 0–0.11 g and 0.06–18.68% of wet weight and the predicted weight of ovaries was 0–0.62 g and 0–39.33% of wet weight. Predicted lipid weight and ovary weight were significantly correlated with body condition ratio (lipid: n = 222, r^2^ = 0.36, p<0.01, ovary: n = 222, r^2^ = 0.87, p<0.01). The correlation between ovary weight and body size was also significant but comparatively low (n = 222, r^2^ = 0.28, p<0.01). There was no correlation between the month of sampling and the size (n = 222, r^2^<0.01, p = 0.91) or weight (n = 222, r^2^ = 0.013, p = 0.10) of spiders.

### c) Landscape scale

**Figure 1:**
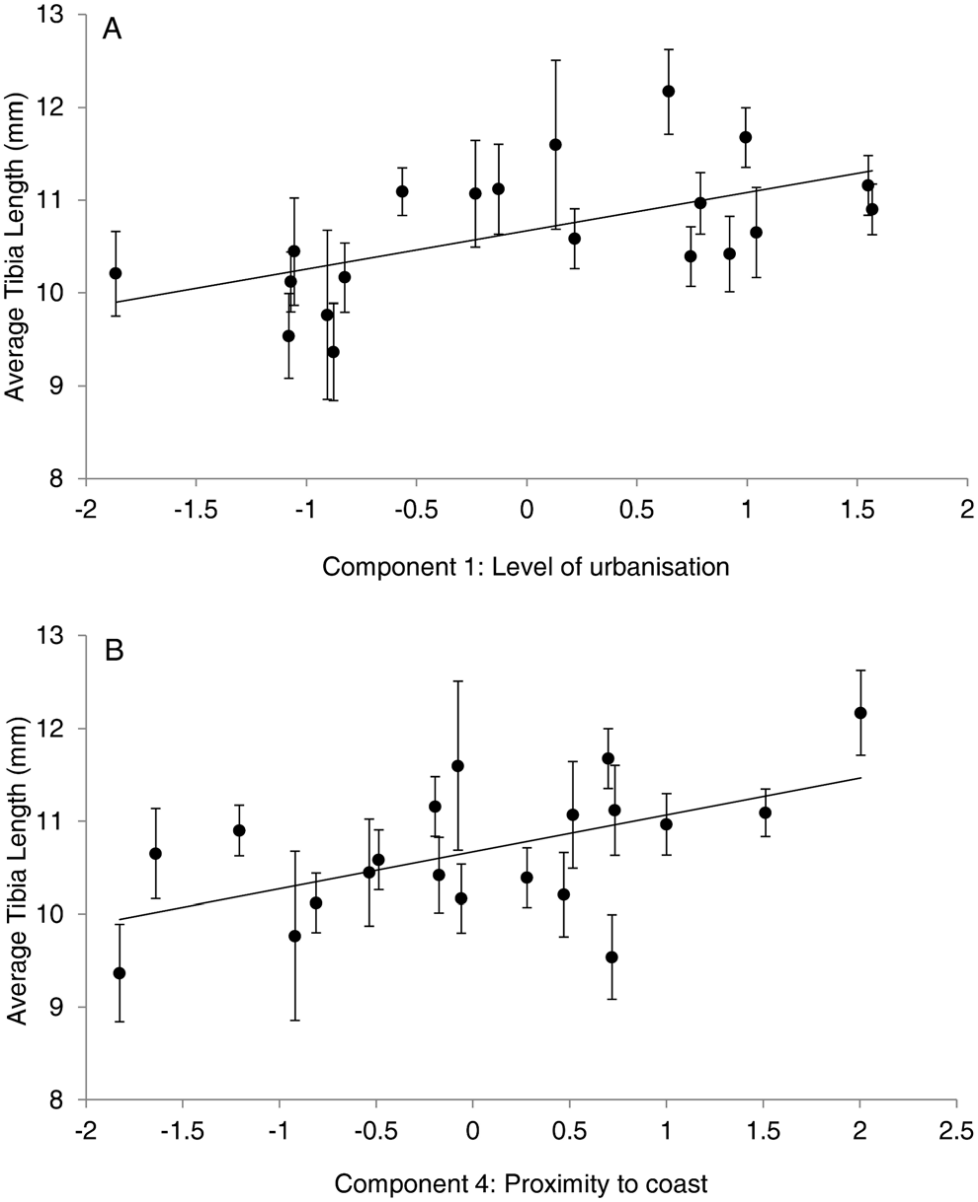
Relationships (site average +/− S.E) between the factorials of landscape scale components and spider size. a) Positive correlation between average tibia length and landscape component 1: positive association with hard surface land cover, housing density, distance to bush land and population density and a negative association with vegetation land cover and distance to CBD, (n = 20, r^2^ = 0.33, p<0.01). b) Positive correlation between average tibia length and landscape component 4: positive association with percentage water cover and negative association with distance to the coastline (n = 20, r^2^ = 0.30, p = 0.01).

When sites were analysed using a categorical predictor (park, remnant or bushland) no significant differences in size (F_2,17_ = 2.9, *p* = 0.07) or condition (ratio: F_2,17_ = 1.19, *p* = 0.32; lipid: F_2,17_ = 0.22 *p* = 0.80; ovary: F_2,17_ = 1.19, *p* = 0.32) were found between areas. However when sites were quantified using the four components from the landscape scale PCA, significant correlations were found between tibia length and components 1 (urbanisation axis) and 4 (proximity to coast, Figure 1), and between ovary weight and component 2 (socioeconomic axis) (n = 20, r^2^ = 0.20, p = 0.05). Lipid weight was not associated with any components from the landscape PCA. Spiders were significantly larger in sites closer to CBD and the coast, with less vegetation and more grass cover (Table 1a). Larger spiders in better condition were found in suburbs with higher socioeconomic status (higher average income, populations and housing density) (Table 1a).

**Table 1:** Pearson R correlations between landscape variables at multiple scales and spider morphology (*P<0.05, **P<0.01).

		Size	Condition ratio	Lipid weight	Ovary weight
Landscape scale	Distance to CBD	−0.51*	−0.44	−0.39	−0.49*
	Distance to water	−0.13	−0.08	0.11	−0.05
	Distance to coast	−0.49*	−0.19	−0.07	−0.24
	Distance to park	−0.43	−0.31	−0.20	−0.38
	Distance to bushland	0.34	0.22	0.26	0.19
	1 km radius % vegetation	−0.62**	−0.37	−0.28	−0.44
	1 km radius % grass	0.47*	0.22	−0.01	0.28
	1 km radius % hard surface	0.36	0.27	0.20	0.28
	1 km radius % water	0.26	0.05	0.12	0.15
	Suburb population density	0.66**	0.55*	0.58**	0.61**
	Suburb housing density	0.66**	0.59**	0.59**	0.63**
	Suburb weekly income	0.19	0.51*	0.56*	0.52*
Local scale	Site % vegetation	−0.42	−0.30	−0.18	−0.34
	Site % grass	0.38	0.29	0.08	0.29
	Site % hard surface	0.57**	0.46*	0.36	0.54*
	Site % buildings	0.24	0.05	0.18	0.14
	Site % water	−0.07	0.03	−0.15	0.02
	Perimeter (m)	−0.51*	−0.36	−0.35	−0.43
	Site Area (m^2^)	−0.39	−0.38	−0.36	−0.44
	Site shape	0.50*	0.58**	0.37	0.58**
Microhabitat scale	Tree canopy cover	−0.12	−0.06	−0.02	−0.05
	Shrub canopy cover	0.13*	0.15*	0.10	0.10
	Ground vegetation cover	−0.01	0.01	−0.02	0.01
	Litter logs and rocks	−0.07	−0.15*	−0.13	−0.14*
	Tree density	−0.03	0.05	0.10	0.08
	Anthropogenic structures	0.14*	0.05	0.06	0.05
	Total habitat quality	−0.08	<0.01	0.01	<0.01
	10 m % veg	−0.09	−0.04	0.01	−0.02
	10 m % hard surface	0.13*	−0.01	0.04	−0.01
	10 m % grass	0.01	0.12	0.01	0.08
	10 m % water	−0.10	−0.06	−0.04	−0.05
	Distance to open space	−0.14*	−0.04	0.04	−0.04
	Distance to hard surface	−0.18*	−0.06	0.04	−0.06
	Distance to building	−0.12	−0.09	0.01	−0.11
	Distance to edge	−0.12	−0.07	0.04	−0.08

Correlations are shown between landscape (n = 20) and local scale variables (n = 20) and the site average size and condition measures. Microhabitat variables are compared to individual size and condition measurements (n = 222).

### d) Local scale

**Figure 2:**
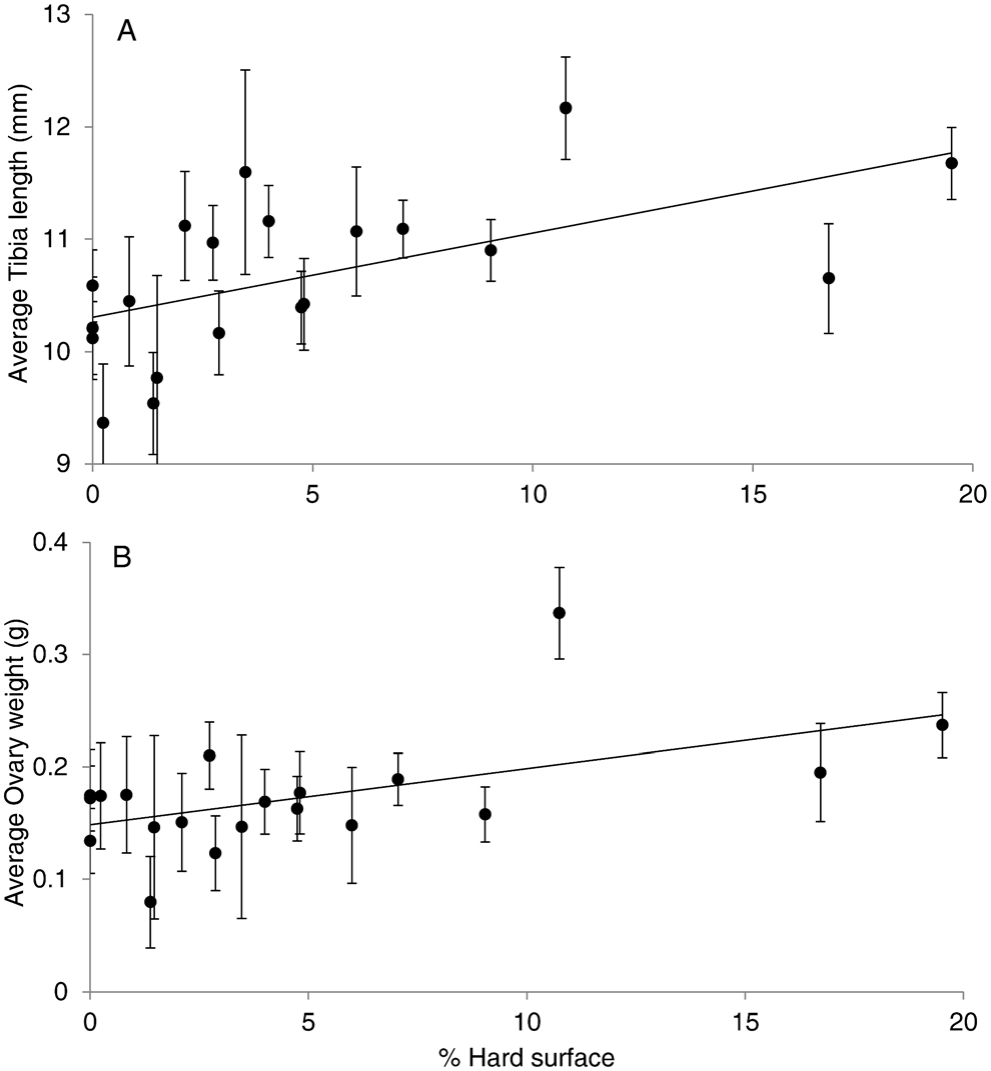
Relationships (site average +/− S.E) between the percentage of each site covered in hard surfaces and spider morphology. a) Positive correlation between percentage hard surfaces and the average tibia length (r^2^ = 0.33, p<0.01). b) positive correlation between the percentage hard surface and average ovary weight (r^2^ = 0.30, p = 0.01).

The first component of the local PCA showed no correlations with any of the morphological traits. The second component (associated with larger, less elongated sites) showed significantly negative correlations with tibia length (r^2^ = 0.23, p = 0.03), condition ratio (r^2^ = 0.20, p = 0.05) and ovary weight (r^2^ = 0.23, p = 0.03), meaning that smaller and convoluted sites were associated with larger spiders. When analysing the local variables independently, tibia length, condition ratio and ovary weight were positively correlated to the percentage of the site covered by hard surfaces (Figure 2) and the shape of the site (Table 1b).

### e) Microhabitat scale

Spiders were larger in microhabitats close to anthropogenic disturbance (described by Component 1, Table 2) and in microhabitats with less natural vegetation and more hard surfaces (Component 2, Table 2). In microhabitats with less leaf litter cover, spiders had higher condition and ovary weights (Table 1c).

**Table 2:** Pearson’s R correlations between the components of the microhabitat PCA, size and microhabitat variables (*P<0.05, **P<0.01).

	Microhabitat PCA component				
	1	2	3	4	5
Tibia length	−0.13*	−0.14*	0.01	−0.03	0.09
Web area	0.12	0.17*	−0.04	0.11	−0.05
Number of Kleptoparasites	0.02	0.14*	−0.02	−0.06	−0.05
Spiders in aggregation	−0.06	−0.11	−0.24**	0.11	0.36**
Prey stored in web	0.16*	0.03	−0.19**	0.05	−0.08

1 = Increased distance to urban features (hard surface, open space, buildings, site edge), 2 = More natural habitat (less hard surfaces and man-made structures, more vegetation) 3 = Increased ground cover complexity (less grass, more undergrowth), 4 = Increased tree and canopy complexity, 5 = Increased mid layer vegetation complexity.

There were also correlations between microhabitat variables and the characteristics of spider webs, kleptoparasite numbers and the size of spider aggregations. Larger webs and increased numbers of kleptoparasites were found in areas with less hard surfaces and man-made objects and more vegetation (Component 2, Table 2). The size of the web and the number of kleptoparasites were not correlated (n = 203, r^2^<0.01, p = 0.71) but spiders in larger webs had increased lipid weight (r^2^ = 0.03, p<0.01), ovary weight (r^2^ = 0.02, p = 0.05) and tibia length (r^2^ = 0.04, p<0.01).

Spiders formed larger aggregations in areas with less ground cover complexity (Component 3, Table 2) and more ground and vegetation cover (Component 5, Table 2). Prey was stored more often by spiders located far from urban disturbance (Component 1, Table 2) but less prey was stored in habitats with increased leaf litter and less grass (Component 3, Table 2). Ovary weight was positively correlated with the number of kleptoparasites in the web (r^2^ = 0.02, p = 0.03), stored prey (r^2^ = 0.03, p<0.01) and number of aggregating spiders (r^2^ = 0.02, p = 0.03).

## Discussion

Our results show support for our first hypothesis that urbanisation has a positive effect on *N.plumipes* body size and ovary weight at multiple landscape scales. Spider size increased with hard surface cover at a local and microhabitat scale and decreased in areas with more vegetation cover. Spider ovaries were significantly larger in sites in close to the city, and in areas with more hard surfaces and less leaf litter. Our second hypothesis was that microhabitat, rather than landscape scale urbanisation would have the largest effects on spider growth and development. Contrary to this, our results show that spider size mostly related to urbanisation over large scales, especially loss of vegetation and creation of hard surfaces.

*N.plumipes* may be pre-adapted to become an urban exploiter, as *Nephila* are often edge dwellers and can reach large abundances under suitable conditions such as high prey availability [39]. Other web weaving spiders are also known to thrive in urban environments [40] and it is likely they are responding to similar modifications to the natural environment. This work provides evidence that conditions in urban areas can influence the morphology of spiders and could also affect other fitness measures such as metabolic rate and behaviour. By identifying the elements of cities that influence the success of urban exploiters we gain a better understanding of what drives changes in the biodiversity of urban systems.

The two factors most likely to explain these morphological differences in spiders from urban environments are increased temperature and prey availability. As temperature can have a significant effect on the growth and size of spiders [30], *N.plumipes* may benefit from the heat island effect attributed to urbanisation [13]. The urban heat island effect is primarily a result of hard surfaces and lack of vegetation cover [41] and studies consistently show significantly higher temperatures associated with anthropogenic land cover [42], [43]. Therefore, the presence of larger spiders in sites with more hard surfaces at both local and microhabitat scales could be a result of increased growth under higher temperatures. At a landscape scale, spider size corresponded negatively to vegetation cover but was not correlated with hard surface cover. This suggests that hard surfaces have the most effect on temperature at a local scale, while vegetation cover affects temperatures at a landscape scale.

Increased prey in urban areas can increase population sizes of urban dwelling spiders [40]. As the quality and abundance of prey can affect spider size [31], an increase of prey in urban areas could also explain the larger size and higher fecundity of urban spiders [39]. Fragment edges combine both open and forest habitats and often have increased abundances of arthropods [44], therefore the larger size of spiders in sites with a more elongated shape could indicate increased prey as a result of edge effects. In Sydney small remnant fragments have the same [45] or higher numbers of invertebrate prey than larger fragments [20] but this response may not be universal as a study by Miyashita et al. [46] found reduced size of *Nephila* spiders in smaller urban fragments as a result of lack of prey.

Another explanation for the correlation between larger spiders and elongated sites is increased exposure to surrounding urban light, as large spiders were also associated with anthropogenic structures such as light posts and were larger in sites closer to the CBD. Artificial night lighting has many implications for spider fitness as it leads to local increases in insect abundance [47], and increased prey capture for spiders in lit habitats [48]. Identifying the important prey for *N. plumipes* and an investigation into how prey varies in relation to urbanisation would provide a more direct test of this hypothesis.

We found that spider condition was negatively associated with leaf litter and positively associated with housing density and hard surfaces. Spiders were also more likely to aggregate and store more prey in microhabitats which were dominated by grass instead of leaf litter, indicating higher prey density and more constant encounter rates [49]. This is despite previous studies showing an increase of spider prey availability in microclimates with deeper litter [50] and the fact that the primary prey for *N.plumipes* (Coleoptera, Diptera, Lepidoptera and Hymenoptera [51]) prefer areas with leaf litter [52], [53]. It is possible that refuse from houses results in higher prey numbers [54] by providing a richer food source for prey arthropods (e.g., flies) than leaf litter. The higher numbers of prey found in a dessert urban system showed no association with increased condition of an urban spider [55] but this trend could differ in more temperate ecosystems.

Our results showed increased numbers of kleptoparasites in areas with less hard surfaces and in webs associated with vegetation rather than anthropogenic structures, as well as supporting previous studies that found an increase in kleptoparasite number with web size [56]. The higher numbers of kleptoparasites in webs further from urban disturbance supports previous studies which have found a negative association between parasite abundance and urbanisation [11] or habitat health [57]. This also demonstrates the differential effect of urbanisation on spiders with different foraging strategies. As kleptoparasites have been shown to negatively affect *Nephila sp.* size [58], the reduced parasite burden in urban areas could be contributing to the larger size of urban spiders.

On a local scale, our study found that spiders were larger, had heavier ovaries and increased lipid storage in suburbs with higher population densities and higher average household income. This increase in ovary size relates to both the larger size of the spiders and the increase in condition. Reproductive output of spiders can increase in response to optimal habitat [59], indicating that high density, wealthy urban areas could be more beneficial habitat for *N.plumipes* than less urbanised areas. Socioeconomics can have a significant effect on biodiversity, with plant and animal species richness shown to increase with household income in urban areas [60]. The increased expenditure and management of parks in wealthy suburbs could result in healthier vegetation patches [61], which would increase prey abundance and allow spiders to grow larger and build up fat reserves.

The positive correlation between ovary weight and urbanisation at local and landscape scales, indicates that urban spiders were at a more advanced stage of reproduction at the time of collection. Anthropogenesis has been shown to change the life history strategies of urban dwellers, for example altering the timing of reproduction in birds [62] and the reproductive capacity of butterflies [63]. As developmental plasticity has been demonstrated in *N.plumipes* [64] it is possible that warmer temperatures have allowed females living in urban areas to mature earlier and mate sooner than females from rural populations. This would present a significant reproductive advantage by allowing the production of multiple egg sacks [65] and may partially explain the success of these spiders in urban landscapes.

Our study shows that *N.plumipes* is an urban exploiter, as size increases with urbanisation at all scales and fecundity, lipid reserves and condition were positively related to a range of anthropogenic changes at one or more scales. The fact that these trends differ according to spatial scale and were not apparent when sites were coarsely categorised highlights the importance of investigating multiple components of urban modification rather than using landscape scale evaluations. While organismal and behavioural studies are often concerned with direct habitat associations, and ecologists factor in broad scale associations, it is rare for both to be considered in the same study. The variation in traits such as land cover between sites of the same coarse landscape categories reveal the heterogeneity of urban systems and emphasises the need for replication of sites within urban areas. When investigating the effects of urbanisation future research should incorporate both microhabitat and landscape scale environmental variables in order to determine the mechanisms driving changes to the morphology of urban wildlife.

## Supporting Information

Table S1 **Urbanisation index, site area and the number of spiders collected for all field sites arranged by coarse landscape category.**
(PDF)

Table S2 **Components and their associated variables for the PCA at landscape, local and microhabitat scales.**
(PDF)

Table S3 **Morphological and microhabitat data.**
(PDF)

## References

[1] DeFries RS, Rudel T, Uriarte M, Hansen M (2010) Deforestation driven by urban population growth and agricultural trade in the twenty-first century. Nature Geosci 3: 178–181.

[2] Shochat E, Warren PS, Faeth SH, McIntyre NE, Hope D (2006) From patterns to emerging processes in mechanistic urban ecology. Trends in Ecology & Evolution 21: 186–191. 16701084 10.1016/j.tree.2005.11.019

[3] Faeth SH, Warren PS, Shochat E, Marussich WA (2005) Trophic Dynamics in Urban Communities. BioScience 55: 399–407.

[4] Grimm NB, Faeth SH, Golubiewski NE, Redman CL, Wu J, et al (2008) Global Change and the Ecology of Cities. Science 319: 756–760. 18258902 10.1126/science.1150195

[5] Blair RB (1996) Land use and avian species diversity along an urban gradient. Ecological Applications 6: 506–519.

[6] Hu Y, Cardoso GC (2009) Are bird species that vocalize at higher frequencies preadapted to inhabit noisy urban areas? Behavioral Ecology 20: 1268–1273.

[7] Dominoni DM, Helm B, Lehmann M, Dowse HB, Partecke J (2013) Clocks for the city: circadian differences between forest and city songbirds. Proceedings of the Royal Society B: Biological Sciences 280. 10.1098/rspb.2013.0593 PMC3774226 23740778

[8] Liker A, Papp Z, Bókony V, Lendvai ÁZ (2008) Lean birds in the city: body size and condition of house sparrows along the urbanization gradient. Journal of Animal Ecology 77: 789–795. 18479344 10.1111/j.1365-2656.2008.01402.x

[9] Antonov A, Atanasova D (2003) Small-scale differences in the breeding ecology of urban and rural Magpies *Pica pica* . Ornis Fennica 80: 21–30.

[10] McDonnell M, Hahs A (2008) The use of gradient analysis studies in advancing our understanding of the ecology of urbanizing landscapes: current status and future directions. Landscape Ecology 23: 1143–1155.

[11] Christie F, Cassis G, Hochuli D (2010) Urbanization affects the trophic structure of arboreal arthropod communities. Urban Ecosystems 13: 169–180.

[12] Cadenasso ML, Pickett STA, Schwarz K (2007) Spatial heterogeneity in urban ecosystems: reconceptualizing land cover and a framework for classification. Frontiers in Ecology and the Environment 5: 80–88.

[13] Kalnay E, Cai M (2003) Impact of urbanization and land-use change on climate. Nature 423: 528–531. 12774119 10.1038/nature01675

[14] Parris KM, Hazell DL (2005) Biotic effects of climate change in urban environments: The case of the grey-headed flying-fox (*Pteropus poliocephalus*) in Melbourne, Australia. Biological Conservation 124: 267–276.

[15] Gehrt SD, Chelsvig JE (2003) Bat activity in an urban landscape: patterns at the landscape and microhabitat scale. Ecological Applications 13: 939–950.

[16] Tylianakis JM, Didham RK, Bascompte J, Wardle DA (2008) Global change and species interactions in terrestrial ecosystems. Ecology Letters 11: 1351–1363. 19062363 10.1111/j.1461-0248.2008.01250.x

[17] Gering JC, Blair RB (1999) Predation on Artificial Bird Nests along an Urban Gradient: Predatory Risk or Relaxation in Urban Environments? Ecography: 532–541.

[18] Gaublomme E, Hendrickx F, Dhuyvetter H, Desender K (2008) The effects of forest patch size and matrix type on changes in carabid beetle assemblages in an urbanized landscape. Biological Conservation 141: 2585–2596.

[19] Sorace A, Gustin M (2009) Distribution of generalist and specialist predators along urban gradients. Landscape and Urban Planning 90: 111–118.

[20] Gibb H, Hochuli DF (2002) Habitat fragmentation in an urban environment: large and small fragments support different arthropod assemblages. Biological Conservation 106: 91–100.

[21] Schmitz OJ (2008) Effects of Predator Hunting Mode on Grassland Ecosystem Function. Science 319: 952–954. 18276890 10.1126/science.1152355

[22] Alaruikka D, Kotze DJ, Matveinen K, Niemelä J (2002) Carabid Beetle and Spider Assemblages along a Forested Urban–Rural Gradient in Southern Finland. Journal of Insect Conservation 6: 195–206.

[23] Magura T, Horváth R, Tóthmérész B (2010) Effects of urbanization on ground-dwelling spiders in forest patches, in Hungary. Landscape Ecology 25: 621–629.

[24] Shochat E, Stefanov WL, Whitehouse MEA, Faeth SH (2004) Urbanization and spider diversity: influences of human modification of habitat structure and productivity. Ecological Applications 14: 268–280.

[25] Bolger DT, Beard KH, Suarez AV, Case TJ (2008) Increased abundance of native and non-native spiders with habitat fragmentation. Diversity and Distributions 14: 655–665.

[26] Churchill TB, Arthur JM (1999) Measuring Spider Richness: Effects of Different Sampling Methods and Spatial and Temporal Scales. Journal of Insect Conservation 3: 287–295.

[27] Niemelä J, Kotze DJ, Venn S, Penev L, Stoyanov I, et al (2002) Carabid beetle assemblages (Coleoptera, Carabidae) across urban-rural gradients: an international comparison. Landscape Ecology 17: 387–401.

[28] Harvey MS, Austin AD, Adams M (2007) The systematics and biology of the spider genus Nephila (Araneae: Nephilidae) in the Australasian region. Invertebrate Systematics 21: 407–451.

[29] “Atlas of Living Australia.” Available: http://bie.ala.org.au/species/Nephila+plumipes. Accessed 2014 Jun 19.

[30] Li D, Jackson RR (1996) How temperature affects development and reproduction in spiders: A review. Journal of Thermal Biology 21: 245–274.

[31] Higgins L, Goodnight C (2011) Developmental response to low diets by giant *Nephila clavipes* females (Araneae: Nephilidae). Journal of Arachnology 39: 399–408.

[32] Meineke EK, Dunn RR, Sexton JO, Frank SD (2013) Urban Warming Drives Insect Pest Abundance on Street Trees. PLoS ONE 8: e59687. 23544087 10.1371/journal.pone.0059687 PMC3609800

[33] Sattler T, Borcard D, Arlettaz R, Bontadina F, Legendre P, et al (2010) Spider, bee, and bird communities in cities are shaped by environmental control and high stochasticity. Ecology 91: 3343–3353. 21141195 10.1890/09-1810.1

[34] Barbaro L, Pontcharraud L, Vetillard F, Guyon D, Jactel H (2005) Comparative responses of bird, carabid, and spider assemblages to stand and landscape diversity in maritime pine plantation forests. Ecoscience 12: 110–121.

[35] ABS (2011) Australian Buro of Statistics. Census Community Profiles.

[36] Lassau SA, Hochuli DF, Cassis G, Reid CAM (2005) Effects of habitat complexity on forest beetle diversity: do functional groups respond consistently? Diversity and Distributions 11: 73–82.

[37] Jakob EM, Marshall SD, Uetz GW (1996) Estimating Fitness: A Comparison of Body Condition Indices. Oikos 77: 61–67.

[38] Wilder S, Rypstra A (2010) Males make poor meals: a comparison of nutrient extraction during sexual cannibalism and predation. Oecologia 162: 617–625. 19960354 10.1007/s00442-009-1518-3

[39] Miyashita T (1992) Food limitation of population density in the orb-wed spider, *Nephila clavata* . Researches on Population Ecology 34: 143–153.

[40] Trubl P, Gburek T, Miles L, Johnson J (2012) Black widow spiders in an urban desert: Population variation in an arthropod pest across metropolitan Phoenix, AZ. Urban Ecosystems 15: 599–609.

[41] Yuan F, Bauer ME (2007) Comparison of impervious surface area and normalized difference vegetation index as indicators of surface urban heat island effects in Landsat imagery. Remote Sensing of Environment 106: 375–386.

[42] Lo CP, Quattrochi DA, Luvall JC (1997) Application of high-resolution thermal infrared remote sensing and GIS to assess the urban heat island effect. International Journal of Remote Sensing 18: 287–304.

[43] Dousset B, Gourmelon F (2003) Satellite multi-sensor data analysis of urban surface temperatures and landcover. ISPRS Journal of Photogrammetry and Remote Sensing 58: 43–54.

[44] Bolger DT, Suarez AV, Crooks KR, Morrison SA, Case TJ (2000) Arthropods in urban habitat fragments in southern California: area, age, and edge effects. Ecological Applications 10: 1230–1248.

[45] Emery TJ, Emery DL (2004) Insect biodiversity in three Sydney urban parklands with differeing levels of human usage. In: Lunney D, S Burgin, editors. Urban Wildlife: More than meets the eye. Mosman, NSW: Royal Zoological Society of New South Wales. 124–130.

[46] Miyashita T, Shinkai A, Chida T (1998) The effects of forest fragmentation on web spider communities in urban areas. Biological Conservation 86: 357–364.

[47] Eisenbeis G, Hänel A, McDonnell M, Hahs A, Breuste J (2009) Light pollution and the impact of artificial night lighting on insects. Ecology of cities and towns: a comparative approach Cambridge University Press, New York, New York, USA: 243–263.

[48] Heiling AM (1999) Why Do Nocturnal Orb-Web Spiders (Araneidae) Search for Light? Behavioral Ecology and Sociobiology 46: 43–49.

[49] Griffiths BV, Holwell GI, Herberstein ME, Elgar MA (2003) Frequency, composition and variation in external food stores constructed by orb-web spiders: *Nephila edulis* and *Nephila plumipes* (Araneae: Araneoidea). Australian Journal of Zoology 51: 119–128.

[50] Uetz GW (1979) The influence of variation in litter habitats on spider communities. Oecologia 40: 29–42. 28309601 10.1007/BF00388808

[51] Herberstein ME, Elgar MA (1994) Foraging strategies of Eriophora transmarina and *Nephila plumipes* (Araneae: Araneoidea): Nocturnal and diurnal orb-weaving spiders. Australian Journal of Ecology 19: 451–457.

[52] Koivula M, Punttila P, Haila Y, Niemelä J (1999) Leaf litter and the small-scale distribution of carabid beetles (Coleoptera, Carabidae) in the boreal forest. Ecography 22: 424–435.

[53] Dugdale JS (1996) Natural history and identification of litter-feeding Lepidoptera larvae (Insecta) in beech forests, Orongorongo Valley, New Zealand, with especial reference to the diet of mice (*Mus musculus*). Journal of the Royal Society of New Zealand 26: 251–274.

[54] McIntyre NE (2000) Ecology of Urban Arthropods: A Review and a Call to Action. Annals of the Entomological Society of America 93: 825–835.

[55] Johnson JC, Trubl PJ, Miles LS (2012) Black Widows in an Urban Desert: City-Living Compromises Spider Fecundity and Egg Investment Despite Urban Prey Abundance. American Midland Naturalist 168: 333–340.

[56] Agnarsson I (2011) Habitat patch size and isolation as predictors of occupancy and number of argyrodine spider kleptoparasites in Nephila webs. Naturwissenschaften 98: 163–167. 21136246 10.1007/s00114-010-0750-3

[57] Hudson PJ, Dobson AP, Lafferty KD (2006) Is a healthy ecosystem one that is rich in parasites? Trends in Ecology & Evolution 21: 381–385. 16713014 10.1016/j.tree.2006.04.007

[58] Grostal P, Walter DE (1997) Kleptoparasites or Commensals? Effects of *Argyrodes antipodianus* (Araneae: Theridiidae) on *Nephila plumipes* (Araneae: Tetragnathidae). Oecologia 111: 570–574. 28308120 10.1007/s004420050273

[59] Puzin C, Acou A, Bonte D, Petillon J (2011) Comparison of reproductive traits between two salt-marsh wolf spiders (Araneae, Lycosidae) under different habitat suitability conditions. Animal Biology 61: 127–138.

[60] Kinzig AP, Warren P, Martin C, Hope D, Katti M (2005) The effects of human socioeconomic status and cultural characteristics on urban patterns of biodiversity. Ecology and Society 10: 23.

[61] Alvey AA (2006) Promoting and preserving biodiversity in the urban forest. Urban Forestry & Urban Greening 5: 195–201.

[62] Partecke J, Van’t Hof T, Gwinner E (2004) Differences in the timing of reproduction between urban and forest European blackbirds (*Turdus merula*): result of phenotypic flexibility or genetic differences? Proceedings of the Royal Society of London Series B: Biological Sciences 271: 1995–2001. 15451688 10.1098/rspb.2004.2821 PMC1691820

[63] Karlsson B, Van Dyck H (2005) Does habitat fragmentation affect temperature-related life-history traits? A laboratory test with a woodland butterfly. Proceedings of the Royal Society B: Biological Sciences 272: 1257–1263. 16024390 10.1098/rspb.2005.3074 PMC1564113

[64] Kasumovic MM, Bruce MJ, Herberstein ME, Andrade MCB (2009) Evidence for developmental plasticity in response to demographic variation in nature. Ecology 90: 2287–2296. 19739390 10.1890/08-1540.1

[65] Schneider JM, Elgar MA (2002) Sexual cannibalism in *Nephila plumipes* as a consequence of female life history strategies. Journal of Evolutionary Biology 15: 84–91.

