# Supplementary material for: Urbanisation at Multiple Scales Is Associated with Larger Size and Higher Fecundity of an Orb-Weaving Spider
Source: PLoS One. 2014 Aug 20;9(8):e105480. doi: 10.1371/journal.pone.0105480 (PMC4139358; doi:10.1371/journal.pone.0105480)
Supplement: Table S1 — Urbanisation index, site area and the number of spiders collected for all field sites arranged by coarse landscape category. (PDF) [file pone.0105480.s001.pdf]

Table S1: Urbanisation index, site area and the number of spiders collected for all field sites arranged by coarse landscape category.

Landscape type	Site name	Area (km^2)	Urbanisation index	# spiders collected
Park	Castle Cove Oval	8.82	-0.75	2
	David Thomas Reserve	12.55	-0.08	2
	Royal Botanic Gardens	56.51	0.96	16
	St Leonards Park	15.39	0.86	10
	Sydney Park	46.33	0.98	7
	Tamarama Park	4.20	0.72	8
	The University of Sydney	69.63	1.57	22
Remnant	Artarmon	14.72	0.59	16
	Carss Bush Park	27.44	-0.31	5
	Cooper Park	20.06	1.44	16
	Georges River National Park	38.33	-1.15	16
	Girrahween Park	33.17	0.14	16
	North Arm Reserve	65.23	-0.74	5
	Oatley Park	69.37	-0.68	12
	Primrose Park	15.23	0.77	15
	Soldiers Memorial Park	160.03	-0.45	6
	Wellings Reserve	8.32	-0.14	7
Bush	Crommelin Arboretum	10.26	-0.89	8
	Brisbane Waters National Park	3810.01	-0.89	8
	Sydney Harbor National Park	61.11	-0.31	25
