# Supplementary material for: Urbanisation at Multiple Scales Is Associated with Larger Size and Higher Fecundity of an Orb-Weaving Spider
Source: PLoS One. 2014 Aug 20;9(8):e105480. doi: 10.1371/journal.pone.0105480 (PMC4139358; doi:10.1371/journal.pone.0105480)
Supplement: Table S2 — Components and their associated variables for the PCA at landscape, local and microhabitat scales. (PDF) [file pone.0105480.s002.pdf]

Table S2: Components and their associated variables for the PCA at Landscape, local and microhabitat scales. +/- values indicate a positive or negative association with each variable within the component

PCA component	% Variance	Represents	Loadings	Association with variables
Landscape 1	43.3	Urbanisation	-0.86	1km % vegetation,
			0.84	1km % hard surface
			0.83	Housing density
			0.80	Population density
			0.78	Site distance to bushland
			-0.77	Site distance to CBD
Landscape 2	13.9	Socioeconomics	0.84	Site distance to parkland
			-0.83	Average house hold income
Landscape 3	12.4	Less grass, close to water	-0.53	1km % grass
			0.84	site distance to water
Landscape 4	9.0	Proximity to coast	-0.77	Site distance to coast
			0.61	1km % water
Local 1	42.1	Urbanisation	-0.96	Site % vegetation
			0.77	Site % grass
			0.76	Site % hard surfaces
			0.71	Site % buildings
Local 2	26.4	Larger and less elongated sites	0.89	Perimeter (m)
			0.84	Site Area (m2)
			-0.80	Shape
			0.42	Site % water
Microhabitat 1	27.5	Further from urban disturbance	0.94	Distance to hard surface
			0.92	Distance to open space
			0.79	Distance to edge
			0.76	Distance to buildings
			0.66	10m %water
Microhabitat 2	17.4	Less urbanised microhabitat	-0.92	10m % hard surfaces
			-0.84	Man-made structures
			0.75	10m % vegetation
Microhabitat 3	10.8	Groundcover complexity	-0.89	10m % grass
			0.61	Leaf litter
Microhabitat 4	8.5	Habitat complexity	0.83	Tree density
			0.63	Total habitat quality
			0.60	Tree canopy cover
Microhabitat 5	8.0	Vegetation density	0.77	Ground vegetation cover
			0.61	Distance to water
			0.47	Shrub cover
