# Supplementary material for: Urbanisation at Multiple Scales Is Associated with Larger Size and Higher Fecundity of an Orb-Weaving Spider
Source: PLoS One. 2014 Aug 20;9(8):e105480. doi: 10.1371/journal.pone.0105480 (PMC4139358; doi:10.1371/journal.pone.0105480)
Supplement: Table S3 — Morphological and microhabitat data. (PDF) [file pone.0105480.s003.pdf]

											Distance to:				
Site	ID	TL	OL	Pw	Wet weight	Predicted lipid weight	Predicted ovary weight	Web area	Prey	Klepto	open space	hard surface	building	edge	water
Usyd	A1	11.78	16.47	5.99	1.19	0.100	4.72		6	0	1	88	140	37	369
Usyd	A2	10.28	14.74	5.74	0.86	0.093	8.69	728.0	9	0	6	6	38	6	232
Usyd	A3	10.44	11.84	5.04	0.46	0.077	0.17	1593.0	8	0	6	6	38	6	232
Usyd	A4	8.08	9.76	4.42	0.24	0.071	10.16	924.0	3	1	6	6	38	6	232
Usyd	A5	12.81	18.38	6.39	1.96	0.122	2.78	938.0	7	1	1	1	48	42	263
Usyd	A6	12.25	15.2	6.09	1.03	0.093	6.26	1410.0	6	0	1	8	39	40	251
Usyd	A7	11.83	15.23	5.76	1.02	0.094	11.00	1563.5	5	1	1	4	39	64	253
Usyd	A8	11.64	12.7	5.77	0.56	0.079	6.26	1787.5	7	0	1	4	39	64	253
Sydney Park	B1	10.1	16.51	5.04	1.22	0.108	0.17	1453.5	2	2	1	1	29	75	192
Sydney Park	B3	9.47	13.83	4.26	0.60	0.085	7.87	1400.0	14	0	1	1	24	77	200
Sydney Park	B4	10.62	18.01	5.53	1.60	0.120	-0.37	1595.0	25	5	1	1	24	77	200
Sydney Park	B5	9.2	13.39	4.38	0.59	0.085	0.17	2080.0	11	9	1	1	24	77	200
Sydney Park	B6	11.48	17.07	5.67	1.21	0.102	0.17	2346.0	1	5	1	1	24	77	200
Sydney Park	B7	10.92	13.31	5.51	0.61	0.082	0.17				8	11	26	103	110
Sydney Park	B8	12.76	17.64	6.93	1.36	0.103	1.53	2320.0	3	3	8	11	26	103	110
Tamarana	C1	12.35	14.52	6.3	0.97	0.091	0.17	855.0	1	0	8	11	26	103	110
Tamarana	C2	12.78	17.96	6.48	1.62	0.111	0.17	1416.0	0	0	6	6	76	66	161
Tamarana	C3	12.81	17.1	6.78	1.48	0.106	0.17	1000.0	0	2	77	77	61	77	47
Tamarana	C4	12.97	16.5	6.85	1.29	0.100	5.27	1860.0	4	1	77	77	61	77	47
Tamarana	C5	12.06	17.35	6.28	1.47	0.109	6.98	1400.0	2	0	70	70	52	70	66
Tamarana	C6	13.03	18.82	6.61	1.98	0.121	0.17	2697.5	4	1	70	70	52	70	66
Tamarana	C7	11.94	21.18	7.19	2.63	0.149	0.17	1292.5	14	0	70	70	52	70	66
Tamarana	C8	9.39	18.5	5.53	1.75	0.135	4.62	1288.0	36	0	1	24	87	71	408
Chowder Bay	D1	9.88	9.32	5.24	0.31	0.072	8.63	390.0	1	0	15	24	430	73	73
Chowder Bay	D10	11.75	14.47	6.39	0.91	0.091	5.15	1563.5	2	3	3	14	44	114	1705
Chowder Bay	D11	11.83	17.44	6.19	1.51	0.111	0.17	1708.0	4	2	70	141	87	73	255
Chowder Bay	D12	11.62	18.7	6.4	1.63	0.116	9.56	1127.0	0	5	155	224	172	155	280
Chowder Bay	D13	10.57	12.37	5.48	0.58	0.082	3.60	630.0	1	5	155	224	172	155	280
Chowder Bay	D14	11.54	20.52	5.69	2.17	0.136	4.93	663.0	0	4	16	49	28	16	1379
Chowder Bay	D3	13.67	13.32	6.57	0.73	0.081	0.17	663.0	2	1	3	5	377	96	96
Chowder Bay	D4	11.92	18.1	6.08	1.73	0.118	0.17	2001.0	3	4	3	5	377	96	96
Chowder Bay	D5	11.9	15.39	6.16	1.10	0.097	0.17	1175.0	2	3	3	5	377	96	96

											Distance to:				
Site	ID	TL	OL	Pw	Wet weight	Predicted lipid weight	Predicted ovary weight	Web area	Prey	Klepto	open space	hard surface	building	edge	water
Chowder Bay	D7	12.35	15.71	6.22	1.10	0.095	0.17	1176.0	1	7	3	5	377	96	96
Chowder Bay	D8	12.08	20.67	6.68	2.47	0.143	0.17	1830.0	4	4	15	23	43	43	1540
Chowder Bay	D9	10.25	17.43	5.8	1.42	0.115	9.02	1248.0	2	5	46	108	158	103	1647
Primrose Park	E1	10.93	17.19	6.35	1.35	0.110	0.17	1640.0	0	5	1	19	50	91	725
Primrose Park	E10	11.01	15.03	5.42	1.10	0.100	0.17	816.0	2	1	5	11	88	11	1120
Primrose Park	E11	12.47	18.63	6.78	2.09	0.128	0.17	2208.0	3	0	5	11	88	11	1120
Primrose Park	E12	11.38	16.25	5.83	1.02	0.096	0.17	1296.0	3	1	5	11	88	11	1120
Primrose Park	E13	11.94	20.27	6.39	2.54	0.146	0.17	1125.0	5	1	5	11	88	11	1120
Primrose Park	E14	9.91	15.82	4.88	1.10	0.104	0.17	1008.0	6	0	5	11	88	11	1120
Primrose Park	E15	10.03	12.58	4.97	0.64	0.085	0.25	902.0	2	1	1	4	60	4	1377
Primrose Park	E2	9.29	11.93	4.91	0.50	0.081	2.55	1675.0	3	0	1	19	50	91	725
Primrose Park	E3	11.62	17.42	6.33	1.58	0.115	0.17	2808.0	7	12	1	19	50	91	725
Primrose Park	E4	11.31	16.17	5.99	1.29	0.106	0.17	1770.0	2	0	1	1	63	84	704
Primrose Park	E5	13.2	15.67	6.37	1.12	0.094	0.17	1953.0	3	10	1	1	55	81	710
Primrose Park	E6	12.55	15.49	6.77	1.00	0.092	11.93	1458.0	10	1	1	6	66	92	703
Primrose Park	E7	8.81	11.23	4.63	0.32	0.074	3.70	400.0	2	0	19	6	90	86	676
Primrose Park	E8	10.9	15.89	5.52	1.23	0.105	0.17	2016.0	2	5	5	11	88	11	1120
Primrose Park	E9	9.14	10.47	4.99	0.30	0.073	3.24	1298.0	4	3	5	11	88	11	1120
Oatley	F1	10.05	15.04	4.96	0.87	0.094	0.17	2112.0	26	2	453	398	294	489	489
Oatley	F10	11.09	14.91	5.69	0.83	0.090	0.17	1710.0	6	1	236	236	278	236	344
Oatley	F11	10.64	14.41	5.44	0.84	0.091	10.59	2047.5	11	1	236	236	303	236	349
Oatley	F12	8.88	10.84	4.37	0.34	0.075	0.17	253.0	1	0	231	213	342	231	350
Oatley	F13	13.09	15.8	6.51	1.05	0.092	10.00	2336.0	1	3	230	230	370	230	329
Oatley	F14	10.83	13.25	5.04	0.74	0.087	7.47	1567.5	5	0	213	213	372	213	306
Oatley	F15	8.46	13.78	5.46	0.74	0.095	0.17	1225.0	1	5	213	213	372	213	306
Oatley	F3	9.24	12.8	5.01	0.60	0.086	4.56	2412.0	2	5	438	493	376	438	438
Oatley	F4	7.64	9.97	3.6	0.25	0.072	13.54	1219.0	10	0	407	574	430	407	407
Oatley	F5	10.43	15.92	5.46	1.09	0.102	0.17	1595.0	12	2	345	588	535	345	345
Oatley	F8	10.08	13	5.06	0.66	0.086	0.17	1456.0	0	6	159	159	192	159	401
Oatley	F9	11.56	14.15	6.23	0.86	0.089	0.98	1944.0	8	9	215	215	247	215	365
Lindfield	G1	7.3	14.05	4.06	0.69	0.098	6.83	738.0	2	0	153	55	83	83	719
Lindfield	G2	9.01	10.64	4.4	0.35	0.075	13.11	350.0	0	0	2	5	14	43	747

											Distance to:				
Site	ID	TL	OL	Pw	Wet weight	Predicted lipid weight	Predicted ovary weight	Web area	Prey	Klepto	open space	hard surface	building	edge	water
Lindfield	G3	10.94	13.56	5.41	0.78	0.088	4.18	1982.5	0	0	1	96	129	138	813
Lindfield	G4	8	14.09	4.17	0.74	0.097	5.24		10	-	1	96	129	138	813
Lindfield	G5	12.31	18.46	6.54	1.99	0.125	4.66		1	-	276	176	117	416	570
Lindfield	G6	8.63	14.21	4.73	0.82	0.098	4.65		11	-	453	398	294	489	489
Cooper Park	H1	13.86	18.64	7.53	1.81	0.112	0.17	2502.5	0	6	1	10	44	26	1448
Cooper Park	H10	10.77	14.81	5.77	0.99	0.097	0.17	2184.0	0	3	5	5	12	108	697
Cooper Park	H11	11.84	17.07	6.39	1.42	0.108	0.17	2387.0	1	6	8	8	5	60	646
Cooper Park	H12	10.68	14.33	5.88	0.73	0.087	0.17	906.5	1	17	8	8	5	60	646
Cooper Park	H13	11.01	16.41	5.83	1.24	0.105	3.92	1375.0	0	8	8	8	5	60	646
Cooper Park	H14	12.04	13.96	8.52	0.85	0.088	0.17	1681.5	1	0	1	1	99	78	2500
Cooper Park	H15	10.65	13.8	5.68	0.76	0.088	0.17	1540.0	1	3	34	34	52	52	2500
Cooper Park	H16	12.44	17.85	6.34	1.73	0.116	0.17	544.0	0	9	62	62	10	10	2500
Cooper Park	H2	10.36	13.99	4.67	0.68	0.086	6.20	1820.0	0	2	2	2	10	85	295
Cooper Park	H3	10.94	15.47	6.42	0.76	0.087	4.78	756.0	0	4	2	2	10	85	295
Cooper Park	H4	11.23	17.66	5.3	1.36	0.108	0.17	1198.5	2	9	2	2	10	85	295
Cooper Park	H5	11.02	13.23	5.58	0.73	0.086	0.17	1198.5	0	12	1	2	10	121	561
Cooper Park	H6	10.77	17.67	5.56	1.48	0.115	0.17	902.0	6	43	3	3	14	217	573
Cooper Park	H7	9.8	13.92	4.92	0.66	0.086	10.01	1403.0	1	1	14	1	24	315	688
Cooper Park	H8	10	12.96	5.74	0.66	0.086	11.24	1118.0	3	2	14	1	24	315	688
Cooper Park	H9	11.12	14.38	5.75	0.71	0.085	0.17	1426.0	1	3	1	1	21	213	675
Usyd	I1	11.41	15.7	6.01	1.18	0.101	0.17	1350.0	2	2	1	4	39	64	253
Usyd	I10	10.42	13.66	5.21	0.71	0.087	4.96	1380.0	0	0	13	23	34	13	13
Usyd	I11	12	13.07	5.93	0.78	0.086	2.71	2336.0	0	0	31	38	36	36	47
Usyd	I2	12.99	12.69	6.38	0.65	0.080	0.17		0	0	1	1	62	66	276
Usyd	I3	10.63	14.15	5.32	0.75	0.088	0.17	1920.0	1	0	1	8	29	200	82
Usyd	I4	11.93	13.67	6.15	0.83	0.087	0.17	1081.0	7	0	1	8	29	200	82
Usyd	I5	12.02	15.18	5.74	0.93	0.091	0.17	2584.0	0	0	1	1	72	212	100
Usyd	I6	10.17	13.42	4.89	0.76	0.089	0.17	1612.0	5	0	1	1	24	133	133
Usyd	I8	7.72	8.94	3.88	0.20	0.070	3.01	1274.0	0	0	1	1	49	189	163
Usyd	I9	10.09	14.39	5.54	0.90	0.095	0.17	1372.0	7	0	14	1	40	200	86
Artarmon	J1	11.79	16.69	5.97	1.25	0.102	0.17	2414.0	3	0	7	26	28	79	94
Artarmon	J10	11.64	14.54	5.96	0.79	0.087	4.57	1881.0	5	1	15	57	27	27	342

											Distance to:				
Site	ID	TL	OL	Pw	Wet weight	Predicted lipid weight	Predicted ovary weight	Web area	Prey	Klepto	open space	hard surface	building	edge	water
Artarmon	J12	12.12	15.87	5.41	1.12	0.097	0.17		0	0	20	60	31	31	405
Artarmon	J13	9.94	14.63	4.96	0.86	0.094	9.01		1	0	20	60	31	31	405
Artarmon	J14	10.18	14.09	4.76	0.83	0.092	7.21	1863.0	7	2	6	51	18	34	331
Artarmon	J15	7.39	10.88	3.59	0.34	0.078	0.17	1080.0	8	4	1	5	18	27	271
Artarmon	J16	10.12	13.15	4.92	0.75	0.089	0.17	1350.0	5	0	1	5	18	27	271
Artarmon	J17	9.67	14.24	5	0.73	0.090	10.12	1563.5	4	0	1	1	26	51	229
Artarmon	J2	10.97	15.9	6.12	1.16	0.102	0.17	1581.0	3	6	7	26	28	79	94
Artarmon	J3	11.34	17.46	5.91	1.64	0.118	7.88	3037.5	3	0	7	26	28	79	94
Artarmon	J4	11.32	14.68	5.55	0.99	0.095	0.17	2272.0	5	0	1	13	17	142	31
Artarmon	J5	11.1	15.47	5.4	1.11	0.100	5.98	2124.0	3	0	1	3	31	144	38
Artarmon	J6	9.06	12.79	4.46	0.61	0.086	0.17	1392.0	7	1	1	3	31	144	38
Artarmon	J7	10.98	13.99	5.5	0.67	0.084	0.17	2892.5	0	1	1	3	31	144	38
Artarmon	J8	9.13	11.25	4.2	0.36	0.075	0.17	1078.0	0	0	1	3	31	144	38
Artarmon	J9	9.51	15.19	5.21	0.93	0.099	1.77	1219.0	2	0	1	15	24	19	135
Arboretum	K1	10.47	14.93	5.36	1.01	0.099	0.17	1799.5	4	16	1	2	10	121	561
Brisbane Waters	K10	10.4	11.85	4.99	0.52	0.079	2.63	2052.0	3	8	5	31	49	31	469
Brisbane Waters	K11	10.52	14.4	5.03	0.92	0.095	0.17	1652.0	1	11	5	31	49	31	469
Brisbane Waters	K12	10.41	13.53	4.98	0.72	0.087	10.00		0	6	5	31	49	31	469
Brisbane Waters	K13	8.84	12.38	4.07	0.61	0.087	0.17	1248.0	2	2	1	17	40	17	488
Brisbane Waters	K14	7.96	11.66	3.99	0.52	0.086	4.09	1347.5	13	8	1	17	40	17	488
Brisbane Waters	K15	10.67	13.07	4.95	0.50	0.078	8.13	1716.0	1	2	1	17	40	17	488
Brisbane Waters	K16	9.31	10.5	4.69	0.33	0.073	0.93	861.0	0	0	1	17	40	17	488
Arboretum	K2	10.28	14.64	5.15	0.95	0.097	0.17	1620.0	11	19	1	27	36	334	711
Arboretum	K3	11.78	16.45	5.54	1.17	0.100	0.17	1829.0	4	11	5	16	37	361	723
Arboretum	K4	11.68	16.03	5.84	1.15	0.099	0.17	3388.0	1	8	13	2	26	148	671
Arboretum	K5	6.91	11.83	3.66	0.40	0.083	4.14	760.0	6	4	15	2	3	46	378
Arboretum	K6	7.89	10.79	4.02	0.38	0.078	0.17	877.5	3	5	1	3	36	22	254
Arboretum	K7	11.13	15.64	5.38	1.25	0.105	0.17		7	12	16	9	50	9	168
Arboretum	K8	11.52	15.65	5.5	1.09	0.098	0.17	1728.0	1	0	9	11	48	107	151
Brisbane Waters	K9	8.19	11.56	4.25	0.44	0.081	6.50	1904.0	10	5	37	37	88	55	447
Padstow	L1	9.47	14.29	5.03	0.80	0.094	5.37	3204.0	5	18	213	213	372	213	306
Padstow	L10	10.47	14.57	5.52	1.04	0.100	0.17	1921.5	0	3	40	40	82	72	596

											Distance to:				
Site	ID	TL	OL	Pw	Wet weight	Predicted lipid weight	Predicted ovary weight	Web area	Prey	Klepto	open space	hard surface	building	edge	water
Padstow	L11	8.17	11.19	3.67	0.43	0.081	0.17	967.5	1	3	86	109	117	109	545
Padstow	L12	9.54	11.39	4.72	0.39	0.076	3.61	1274.0	0	0	69	106	106	106	494
Padstow	L13	9.02	12.36	4.45	0.55	0.084	8.25	1176.0	2	6	69	82	120	69	561
Padstow	L14	11.13	15.64	5.65	1.24	0.104	0.17		8	6	67	93	131	93	669
Padstow	L15	11.36	13.4	5.27	0.69	0.084	0.17				1	19	50	91	725
Padstow	L16	9.7	14.73	5.09	0.88	0.096	0.17	864.0	4	8	1	19	50	91	725
Padstow	L2	10.48	11.47	5.16	0.50	0.079	10.25	1800.0	4	10	213	213	372	213	306
Padstow	L3	10.47	14.77	5.31	0.99	0.098	0.17	2117.5	10	10	213	213	372	213	306
Padstow	L4	11.62	15.11	6.03	1.03	0.095	14.86	2409.0	0	5	213	213	372	213	306
Padstow	L5	10.29	14.32	4.96	0.94	0.096	0.17	1742.0	11	5	213	213	372	213	306
Padstow	L6	9.51	13.19	4.85	0.63	0.086	0.17		13	7	213	213	372	213	306
Padstow	L7	8.97	14.07	4.61	0.81	0.096	0.17	2600.0	11	3	171	171	447	171	241
Padstow	L8	10.73	14.43	5.23	0.97	0.096	2.86	1971.0	2	5	1	5	48	35	636
Padstow	L9	10.98	14.41	5.43	0.88	0.092	12.88	2232.0	6	11	40	40	82	72	596
Girrahween	M1	11.23	13.65	5.96	0.71	0.085	3.83	2567.5	13	3	5	1	43	43	2500
Girrahween	M10	10.25	17.9	5.14	1.50	0.119	0.52	1344.0	4	4	22	22	43	22	2500
Girrahween	M11	10.46	15.16	5.07	0.84	0.092	5.14	2912.0	9	2	1	4	26	4	2500
Girrahween	M12	10.55	17.43	5.86	1.49	0.117	0.17	1647.0	2	2	1	4	26	4	2500
Girrahween	M13	9.18	12.33	4.34	0.56	0.084	7.82	2047.5	4	1	75	189	117	254	901
Girrahween	M14	7.66	10.91	3.53	0.37	0.079	0.17	987.0	8	5	102	217	212	280	940
Girrahween	M15	13.37	15.57	7.33	1.37	0.101	4.03	3444.5	8	5	153	55	83	83	719
Girrahween	M16	10.78	14.24	5.18	0.93	0.094	0.17	1775.0	11	2	153	55	83	83	719
Girrahween	M2	11.14	15.46	5.14	1.13	0.100	0.17	1809.0	15	7	5	1	43	43	2500
Girrahween	M3	10.39	11.09	5.51	0.41	0.075	1.66	1204.0	1	1	1	1	56	39	2500
Girrahween	M4	10.03	14.38	5.43	0.93	0.097	0.17	3000.0	10	4	1	1	56	39	2500
Girrahween	M5	11.92	16.94	5.69	1.32	0.104	3.41	1272.0	3	1	1	1	56	39	2500
Girrahween	M6	10.96	12.89	5.47	0.61	0.082	1.77	1799.5	2	1	1	1	56	39	2500
Girrahween	M7	9.25	10.92	4.99	0.38	0.076	12.22	2320.0	7	3	6	6	77	53	2500
Girrahween	M8	11.65	15.62	5.74	1.14	0.099	3.77	1708.0	15	2	11	11	70	44	2500
Girrahween	M9	10.52	15.04	5.11	0.95	0.096	0.17	1272.0	5	0	51	51	39	39	2500
St Leonards	N1	12.05	16.06	5.7	1.17	0.099	2.87	1192.5	11	0	1	4	60	4	1377
St Leonards	N10	10.34	14.4	5.44	0.84	0.092	4.78	1512.5	0	0	1	1	29	75	192

											Distance to:				
Site	ID	TL	OL	Pw	Wet weight	Predicted lipid weight	Predicted ovary weight	Web area	Prey	Klepto	open space	hard surface	building	edge	water
St Leonards	N2	10.59	12.85	5.54	0.60	0.082	4.22	1517.0	2	0	1	4	87	4	1414
St Leonards	N3	8.88	12.73	4.51	0.60	0.087	9.22	2077.0	5	0	1	1	26	51	229
St Leonards	N4	8.39	10.87	4.35	0.42	0.080	7.43	882.0	0	0	1	1	26	51	229
St Leonards	N5	10.25	15.21	5.79	0.99	0.099	5.71	967.5	6	0	1	1	26	51	229
St Leonards	N6	10.66	15.82	5.3	1.11	0.101	0.17	1428.0	5	0	1	1	26	51	229
St Leonards	N7	11.26	16.68	5.7	1.39	0.110	0.17	1323.0	7	0	1	1	26	51	229
St Leonards	N8	11.61	16.64	6.88	1.60	0.115	3.89	2437.5	18	0	1	1	40	85	179
St Leonards	N9	10.17	14.13	5.34	0.92	0.096	7.01	352.0	0	0	1	1	40	85	179
Chowder Bay	O1	10.45	9.2	5.12	0.29	0.070	0.17	1081.0	1	3	16	49	28	16	1379
Chowder Bay	O10	12.88	15.66	6.89	1.32	0.101	0.17		1	6	75	73	73	64	1635
Chowder Bay	O11	7.36	8.09	3.53	0.13	0.066	0.17	595.0	3	0	62	63	70	63	1613
Chowder Bay	O12	11.37	16.62	5.74	1.27	0.105	0.17	1400.0	5	3	49	56	62	54	1567
Chowder Bay	O14	11.82	18.02	5.81	1.78	0.121	0.17		11		1	10	49	43	1452
Chowder Bay	O2	11.14	15.26	5.68	1.18	0.102	0.17	2035.5	10	9	16	49	28	16	1379
Chowder Bay	O3	10.85	15.64	5.2	1.10	0.100	0.17	1282.5	8	6	1	60	39	32	1417
Chowder Bay	O4	11.11	14.35	5.59	0.99	0.096	11.89	2257.0	10	0	1	55	40	27	1417
Chowder Bay	O5	10.22	11.81	5.28	0.39	0.075	13.02	756.0	5	1	1	57	37	26	1424
Chowder Bay	O6	8.99	12.98	4.68	0.58	0.086	5.62	1020.0	0	1	8	10	0	46	1460
Chowder Bay	O7	11.95	14.6	5.6	0.86	0.088	11.27	903.0	1	4	8	10	0	46	1460
Chowder Bay	O8	10.35	12.35	5.02	0.53	0.080	8.57	1537.0	17	10	23	23	69	24	1607
Chowder Bay	O9	9.39	10.52	4.7	0.32	0.073	0.17	536.5	4	7	36	29	51	29	1651
Usyd	P1	10.55	16.63	5.98	1.24	0.107	0.17	864.0	11	2	37	28	40	37	44
Usyd	P2	9.67	15.51	5.03	1.02	0.102	0.17	1033.5	18	2	48	20	52	52	54
Usyd	P3	10.21	15.27	5.34	1.06	0.101	5.61	1339.5	29	0	57	24	67	66	67
Usyd	P4	10.85	16.56	6.16	1.26	0.107	7.87	1075.0	14	0	76	32	94	78	78
Castle cove	Q1	11.11	14.13	6.4	0.88	0.091	0.17	797.5	6	7	5	42	267	5	5
Castle cove	Q2	11.88	17.78	6.05	1.68	0.117	0.17	1222.0	4	7	5	42	267	5	5
Castle cove	Q3	10.59	12.62	4.96	0.71	0.087	0.17		2	5	5	47	296	5	5
Castle cove	Q4	9.63	13.44	4.98	0.83	0.094	0.17		6	7	5	47	296	5	5
Castle cove	Q5	9.02	13.15	4.51	0.55	0.084	7.53		3		6	49	347	6	6
Castle cove park	Q6	10.12	13.05	4.87	0.75	0.089	0.17	387.5	4	21	35	4	422	54	54
Castle cove park	Q7	9.41	13.55	4.97	0.80	0.094	5.09	2145.0	2	19	15	24	430	73	73

											Distance to:				
Site	ID	TL	OL	Pw	Wet weight	Predicted lipid weight	Predicted ovary weight	Web area	Prey	Klepto	open space	hard surface	building	edge	water
Botanical Gardens	R1	10.41	15.01	5.14	1.07	0.101	0.17	624.0	0	1	1	1	26	51	229
Botanical Gardens	R10	11.1	15.08	5.46	0.91	0.093	0.17	561.0	0	20	105	52	117	110	110
Botanical Gardens	R11	13.91	17.92	6.95	1.52	0.104	0.17	1380.0	0	8	101	55	46	106	106
Botanical Gardens	R12	14.03	17.42	6.9	1.62	0.106	0.17	882.0	2	2	47	25	88	67	67
Botanical Gardens	R13	12.08	16.81	6.5	1.25	0.101	2.83	1111.5	1	7	47	25	88	67	67
Botanical Gardens	R14	12.5	17.93	6.4	1.93	0.122	0.17	310.5	5	28	21	30	32	69	387
Botanical Gardens	R15	11.51	17.78	6.21	1.46	0.111	0.17	1456.0	2	9	10	5	11	90	377
Botanical Gardens	R16	11.49	15.77	6.19	1.14	0.100	0.17	1057.5	1	17	17	21	69	102	378
Botanical Gardens	R2	10.21	13.99	5.14	0.81	0.092	0.17	684.0	0	6	1	1	26	51	229
Botanical Gardens	R3	10.18	13.18	5.31	0.82	0.092	0.17	777.0	1	1	1	1	26	51	229
Botanical Gardens	R4	10.47	13.76	5.08	0.78	0.090	3.52	814.0	0	17	1	1	40	85	179
Botanical Gardens	R5	12.05	15.85	5.68	1.11	0.097	3.96	1453.5	8	15	1	1	40	85	179
Botanical Gardens	R6	11.54	17.75	5.88	1.77	0.122	0.17	1425.0	8	14	1	1	24	71	192
Botanical Gardens	R7	11.43	15.45	5.21	0.89	0.091	0.17	1175.0	11	19	1	1	24	71	192
Botanical Gardens	R8	11.68	16.79	5.87	1.42	0.109	2.30	742.5	0	10	1	1	24	71	192
Botanical Gardens	R9	12.22	15.15	6.09	0.97	0.091	2.24	1606.5	0	15	50	35	67	130	131
Wellings	S1	12.11	17.48	6.85	1.65	0.115	0.17	713.0	13	14	1	67	74	56	639
Wellings	S2	12.73	15.76	6.94	1.01	0.092	3.25	1296.0	0	15	1	67	74	56	639
Wellings	S3	11.55	13.8	5.62	0.69	0.083	9.57				15	15	74	70	141
Wellings	S4	9.5	12.43	4.89	0.59	0.085	0.17				47	70	56	47	167
Wellings	S5	11.02	13.98	5.5	0.86	0.091	0.17	1260.0	0	15	30	38	27	30	178
Wellings	S6	10.39	13.43	5.13	0.64	0.084	4.37	2124.0	6	13	30	38	27	30	178
Wellings	S7	10.52	12.66	5.05	0.50	0.078	1.14	721.5	1	9	70	72	49	34	228
David Thomas	S8	11.55	12.71	5.57	0.72	0.084	0.17	1296.0	2	6	43	43	9	43	2500
David Thomas	S9	11.64	14.93	6.1	1.05	0.096	4.36	1058.0	1	0	13	13	3	13	2500
Carrs Park	T1	13.91	19.14	6.82	2.00	0.118	3.28	1035.0	2	0	1	4	21	4	495
Carrs Park	T2	11.38	12.84	6.03	0.64	0.082	1.03	666.5	0	4	1	4	21	4	495
Carrs Park	T3	8.85	11.63	4.43	0.36	0.076	0.17	525.0	3	1	1	4	21	4	495
Carrs Park	T4	11.76	15.4	5.57	0.94	0.092	0.17	396.0	0	0	1	11	36	11	457
Carrs Park	T5	9.44	12.46	4.34	0.48	0.080	7.49	576.0	4	2	54	30	44	44	78
